# Ant-plant sociometry in the *Azteca-Cecropia* mutualism

**DOI:** 10.1038/s41598-018-36399-9

**Published:** 2018-12-19

**Authors:** Peter R. Marting, Nicole M. Kallman, William T. Wcislo, Stephen C. Pratt

**Affiliations:** 1 School of Life Sciences, Arizona State University, Tempe, AZ 85281 USA; 2 Smithsonian Tropical Research Institute, Balboa, Panama

## Abstract

A holistic understanding of superorganism biology requires study of colony sociometry, or the quantitative relationships among growth, nest architecture, morphology, and behavior. For ant colonies that obligately nest within plant hosts, their sociometry is likely intertwined with the plant, which has implications for the evolution, strength, and stability of the mutualism. In the *Azteca*-*Cecropia* mutualism, plants provide ants with food rewards and hollow stems for nesting in return for protection from herbivores. Several interesting questions arise when considering ant-plant sociometry: are colony growth and plant growth synchronized? How do colonies distribute themselves within the stem of their host plant? How do plant traits influence worker morphology? How is collective personality related to tree structure, nest organization, and worker morphology? To address these questions, we investigated patterns within and relationships among five major sociometric categories of colonies in the field – plant traits, colony size, nest organization, worker morphology, and collective personality. We found that colony sociometry was intimately intertwined with host plant traits. Colony and plant growth rates were synchronized, suggesting that positive feedback between plant and colony growth stabilizes the mutualism. The colony’s distribution inside the host tree tended to follow leaf growth, with most workers, brood, and the queen in the top half of the tree. Worker morphology correlated with plant size instead of colony size or age, which suggests that plant traits influence worker development. Colony personality was independent of colony distribution and tree structure but may correlate with worker size such that colonies with smaller, less variable workers had more aggressive personalities. This study provides insights into how ant-plant structural relationships may contribute to plant protection and the strength of mutualisms.

## Introduction

To understand how social insect colonies function as superorganisms, it is essential to quantify patterns of colony growth, nest architecture, and morphology, a field of study known as insect sociometry^1^. The relationships and scaling between colony traits give insight about development, collective physiology, evolutionary constraints, and plasticity. Such basic natural history is often scarce or lacks depth because data can be hard to collect.

**Figure 1:**
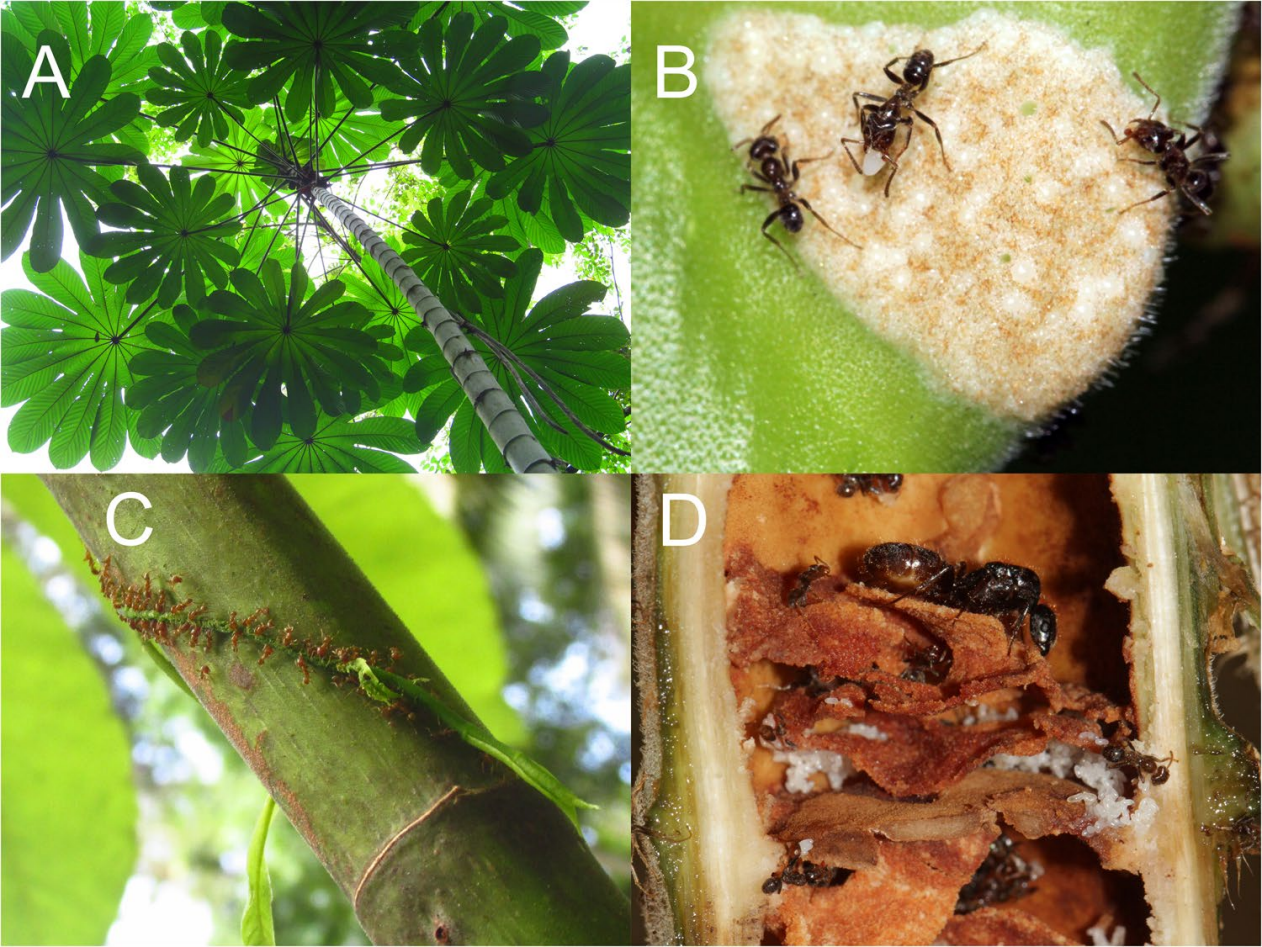
A photographic overview of the *Azteca-Cecropia* mutualism. (**A**) A view from below the crown of a juvenile *Cecropia obtusifolia*. (**B**) *Azteca constructor* havesting Müllerian food bodies from a trichilium. (**C**) *Azteca* workers attacking an enchroching vine. (**D**) A cross-section of the central stem shows the queen, workers, and brood residing in carton galleries inside the hollow internodes. All photos were taken by Peter Marting.

For ant colonies that obligately nest within plant hosts, aspects of their sociometry are likely intertwined with their host plant, which may have interesting implications for the strength and stability of the mutualism. We studied ant-plant sociometry in *Azteca constructor* colonies nesting in *Cecropia* trees in the lowland tropics of central Panama (Fig. 1). *Cecropia* trees provide hollow internodes for nesting and glycogen-rich food bodies for the ants^2,3^, which in return protect the trees from herbivores and vines^4,5^ and provide nitrogen enrichment^6–8^. This system provides a unique and interesting view of insect sociometry because the complex environmental factors that typically shape sociometry – habitat structure, resource abundance, territory size, interactions with intruders, microclimate – are simplified through the colony’s interaction with their host plant. The host plant *is* their environment; a biotic environment possibly shaped by coevolution with the ants themselves (but see^9^). We investigated patterns of and relationships among five major categories of sociometry; tree size, colony size, nest structure, ant morphology, and collective personality. In the following paragraphs, we outline driving questions for each sociometric category through the lens of the mutualism.

*What is the relationship between colony growth and plant growth?* Comparing colony growth rate to that of the host plant reveals potential strains in the mutualism. If plant growth outpaces colony growth, ants may not be able to keep up with herbivory pressure, and plants suffer from leaf damage and a reduction in fitness^10–12^. One possible solution to this problem is the evolution of secondary polygyny through colony budding, where new queens mate intracolonially and do not disperse, allowing the colony to live and grow faster^13,14^. However, *A*. *constructor* display secondary monogyny^15^ where many queens establish the colony together but eventually fight to the death, so this solution is not likely in place. On the other hand, if colony growth outpaces plant growth, the benefit from ant protection diminishes as costs of housing and feeding them increase^16,17^. To avoid such imbalances, growth rates may be equalized by positive feedback between colony and plant growth, reinforcing the mutualism^18^. We can probe these interactions by comparing the scaling coefficients over a large size range, allowing us to estimate how the rate of worker production changes as *A*. *constructor* colonies grow, how the rate of leaf production changes as *Cecropia* trees grow, and whether colonies produce new workers as fast as trees produce new leaves.

*How do colonies structure and organize their nest inside the host plant?* The physical nest architecture of plant-ants is determined by hollow nesting spaces called domatia. Colonies make decisions about which domatia to occupy, how to distribute themselves within the plant, and whether to add structural elements like carton galleries. How a colony is distributed and organized may influence the colonies’ ability to forage, tend brood, respond to threats, and communicate effectively. Little is known about how plant-ant colonies distribute and organize themselves within their host plant, and what forces influence these patterns. The dissection of a large, mature *Cecropia* tree revealed that the majority of the *A*. *constructor* colony is centralized in a large bulge in the main trunk^19^, suggesting that the colony’s distribution may remain static as the tree grows. We investigated a larger sample that includes smaller trees, and measured how colony components – specifically workers, queen, brood, commensal scale insects, refuse piles, carton, and entrances – are distributed in the tree, how these components are spatially related to one another, and how their distribution changes with tree growth.

*How do plants traits influence worker morphology?* Worker size and polymorphism are often associated with sociometric measures, such as colony size, age, and annual cycle^20–23^. Worker morphology within a colony depends on intrinsic factors (genotype and development), external factors (environment and enemies) or a combination of both (nutrition and social environment)^24^. In ant-plant mutualisms, worker morphology might be related to mutualism dynamics or physical traits of the host plants themselves, especially since colony performance feeds back into plant fitness. In the Sonoran desert, ant species with larger body size are associated with more myrmecophyte species^25^, suggesting that they can take advantage of a wider range of resources. A comparison of two plant-ants found that the species with larger body size and greater variation in body size was associated with the host plant species that has larger domatia and prostoma^26^, suggesting that worker morphology may coevolve with plant traits. In addition to plant morphology, worker size may match the size of the dominant herbivores threatening their host. Ant species that invest in smaller workers may be better at scrutinizing the surface of their host plant and removing small sap sucking insects^27^, but worse at fending off larger insects and vertebrates. In addition to plant dimensions, worker morphology may depend on food resources provided to the colony via food bodies^2^ or nutritious pith called parenchyma^28,29^ – plants providing more nutrition may produce larger workers. Morphometric analysis of the non-*Cecropia*-inhabiting congener *Azteca trigona* revealed that workers were dimorphic^30^, but worker size and allometry and their relation to plant traits have not been formally described in *A*. *constructor* until the present study.

*How is colony personality related to tree structure*, *nest organization*, *and worker morphology?* While colony personality is typically independent of colony size^31–34^, colony growth has been correlated with colony behavioral traits^35,36^ or variation therein^33,37^. Sociometric traits beyond colony size and growth likely help shape colony personality and are rarely examined (but see^38^). Colonies of *A*. *constructor* display collective personalities along a docile-aggressive axis for a suite of behavioral traits^34^. The sources of behavioral variation are yet unclear, but are likely to lie at the intersection of genotype and the environment^39,40^. Semi-permanent traits like nest architecture likely effect colony behavior over long periods of time. The physical attributes of nest entrance chambers influence collective behavior by affecting worker encounter rates^41^ or ability to exit the nest in a state of alarm^42^. In the context of an ant-plant mutualism, colony personality and plant traits may be related. Plants provide two major resources for their ant colonies – nesting space and food bodies, both of which are correlated with plant height^43^. Higher resource availability may increase energy reserves, fueling higher activity and aggression^44^. However, the causality may flow in the opposite direction. In the *Azteca*-*Cecropia* mutualism, colonies with more aggressive personalities live in trees with less leaf damage^34^, which may increase plant growth. Finally, colony aggression and plant growth may influence each other in a positive feedback loop, stabilizing the relationship. Colony personality may also interact with worker morphology. While body size and colony behavior were independent in *Temnothorax longispinosus* ants^33^, larger workers of *Cataglyphis niger* ants were more aggressive toward conspecifics in staged encounters^45^. If this trend holds true in *A*. *constructor*, we might expect that at the colony level, colonies with larger average workers are more aggressive.

To address these questions, we harvested trees containing colonies with known personality scores^34^ and measured the number of workers, queen, brood, scale insects, refuse piles, carton, and entrances in each internode to determine how colonies were vertically distributed. We then measured the morphology of a subset of workers from each colony. In addition, we measured key features of host tree morphology, including tree height, diameter, number of internodes, number of leaves, and leaf area.

We first use these data to describe the patterns of each separate sociometric category (plant size, colony size, colony organization, ant morphology, and collective personality), then we explore the relationships among them, focusing on the degree to which colony sociometry is intertwined with host plant biology.

## Methods

### Focal species and study site

*Cecropia* trees are diecious pioneer plants with a single central stem that produces a new hollow, leaf-baring internode every 2–4 weeks^46^. The giant, radial leaves produce Müllerian food bodies at specialized sites called trichilia at the petiole-stem juncture. Leaf lifespan is typically 3–6 months, but food body production peaks a few weeks after the leaf emerges^43^. After 3–5 years, branches grow out from the central stem and bifurcate annually to produce a candelabra structure^47–49^. Workers chew entrances to individual internodes and holes through the septa that separate internodes, creating a nearly complete, internal passageway throughout the length of the tree^19^. Workers can further partition the available volume by constructing carton galleries inside the internodes^50^, made from a combination of regurgitated plant materials including parenchyma, a soft, white tissue lining the inside of newly formed internodes^28^. In a related species, *Azteca brevis*, carton material is structurally reinforced by a multi-species network of fungal hyphae^51^. Dark brown “refuse piles” can be found throughout the internal structure, harboring nematodes^52^ and fungus^29,53^. Colonies display distinct behavioral tendencies, or personalities, in that they differ repeatably in a suite of behavioral traits that are independent of colony size and age^34^.

We located 14 *A*. *constructor* colonies along a 12 km stretch of Pipeline Road in and around the lowland tropical rainforests of Soberania National Park, Colón, Panama between March and May 2013. At this site, there are four common *Cecropia* species (*C*. *peltata*, *C*. *obtusifolia*, *C*. *longipes*, and *C*. *insignis*) and three common *Cecropia*-inhabiting *Azteca* species (*A*. *constructor*, *A*. *alfari*, and *A*. *isthmica*). All pairings of ant and tree species can be found, but *C*. *peltata*, *C*. *longipes*, and *A*. *alfari* tend to be found in large disturbed areas, while the others tend to be found in forest gaps (PM, personal observation) – a trend that may be driven by humidity limitation. For the purposes of this study, we focused on a single *Azteca* species (*A*. *constructor*) that occupied *C*. *obtusifolia* (n = 10), *C*. *peltata* (n = 2), and *C*. *insignis* (n = 2).

Colony founding in *Azteca* involves secondary monogyny, meaning multiple queens cooperate in the incipient stages, and eventually fight to the death until one queen remains^15,29^. To avoid these complex intracolony dynamics, we selected trees old enough to have a single queen (above 2 m tall). Trees can reach over 20 m in height and have many branching points, but we used shorter trees (below 8 m tall) with single stems for assay standardization and ease of access. Therefore, our sampling reflects the sociometry of juvenile trees.

### Tree size

We measured tree height, diameter, and number of leaves upon harvesting the colonies. To assess total leaf area, all leaves were separated, photographed against a light background, and measured using ImageJ software. *Cecropia* internodes have a consistent growth-periodicity internode branching pattern that allows for accurate estimation of plant age^54^: we counted the number of internodes between branching points of larger, mature trees to estimate an average annual internode output for each *Cecropia* species (*C*. *peltata*: n = 11; *C*. *obtusifolia*: n = 10; *C*. *longipes*: n = 10; and *C*. *insignis*: n = 4). We divided the number of internodes from our focal plants by the annual output to estimate plant age. *Azteca* ants colonize *Cecropia* trees as saplings^15^, so while plants are slightly older than colonies, their ages are likely tightly correlated. To estimate the total internal volume of the plant, we measured the internal height and width to calculate the volume of a cylinder (V = πr^2^h) for each internode and summed all cylinders per plant.

### Colony size, nest organization, and vertical distribution

After completing the behavioral trials (described below), we harvested the host trees and extracted entire colonies in August of 2013. To subdue the ants and minimize disturbance to their internal distribution, we used internal and external insecticides in quick succession. The ants chew through most of the internode septa^19^, providing a path for the insecticide to traverse the internal height of the tree. We drilled a hole into the base of each tree and inserted the nozzle of a propane-powered insecticide fogger (active ingredient: resmethrin) and discharged the insecticide for several minutes. The tree was then cut at the base, laid on a large plastic tarp, and sprayed with a liquid insecticide externally (active ingredients: pyrethrins, piperonyl butoxide, and permethrin). While some ants exited the domatia during the harvest, this method provides the best estimate for relative abundance inside the stem. Stems were cut in meter-long segments and split vertically to access the internal colony. For each internode, we quantified the internal domatium dimensions, the number of workers, brood (larvae and pupae not distinguished), scale insects, and refuse piles, and noted the presence of the queen, entrances to the exterior, carton material, and leaf-baring petioles. After we quantified the internal distribution of the colony, we collected all workers from the stems, leaves, tarps, and bags and immediately placed them in 95% ethanol. To survey colony size, workers were spread out on grid paper, photographed, and counted using ImageJ software.

### Ant morphology

For each colony, we selected a subset of 100 workers from a large vial of ethanol containing the entire colony. To reduce size bias selection as much as possible, we mixed the ethanol into a vortex with forceps and selected workers haphazardly. For each ant, we separated head, mesosoma, gaster, and legs, and arranged them on an index card using double-sided tape. With a camera mounted on a dissection scope, we photographed each ant using SPOT imaging software (www.spotimaging.com, Sterling Heights, MI). We calibrated the images with a micrometer scale that was included in each photograph, and measured head width and mesosoma length using ImageJ software.

### Behavioral traits

We related the sociometric measures described above to previous analyses demonstrating collective personalities in these colonies. For detailed methods see^34^, but here we provide a brief description. To characterize colony-level behavior, colonies were subjected to five bioassays: patrolling behavior, vibrational disturbance, response to intruder, response to leaf damage, and exploratory tendency. Colonies received each assay at least two times to assess behavioral consistency (patrolling behavior assay was repeated four times per colony). To standardize behavioral measurements across different tree sizes, we focused on the central stem at the lowest leaf’s internode, which we estimated to be the location of median colony distribution based on four preliminary tree dissections. For patrolling behavior, vibrational disturbance, and response to intruder, we scored activity by counting the number of times we saw a worker completely traverse the lower septum line on the external surface of the focal internode, regardless of direction or identity. For leaf damage assays, we focused on an entire leaf instead of the stem and counted the number of workers on that leaf every minute. Trials were recorded with an HD camcorder (Panasonic HC-X900M) between May and August of 2013.

### Statistical analyses

Data were analyzed with linear correlation and regression, ANOVA, and paired t-tests. We log-transformed colony and tree size variables to evaluate allometric scaling by testing if the observed scaling coefficient (log-log slope) differed from the scaling coefficient predicted in the case of isometry with a Wald test. We square-root-transformed total leaf area before evaluating scaling relationships so that the predicted scaling coefficient for isometry was 1 in all cases. Thus, observed scaling coefficients that were indistinguishable from 1 indicate isometric relationships, below 1 indicate negatively allometry, and above 1 indicate positively allometry.

We used principal component analysis to simplify the characterization of each of the five major categories of sociometrical data (tree size, colony size, colony structure, worker morphology, and colony personality). We performed separate unrotated PCA for each category, to reduce several defining traits to summary variables. Only eigenvalues greater than the mean eigenvalue were used in subsequent analyses^55^. Summary variables were then used to investigate relationships among the categories. All statistical analyses were performed in Stata 12.1.

## Results

### Plant size, colony size, and growth scaling

**Figure 2:**
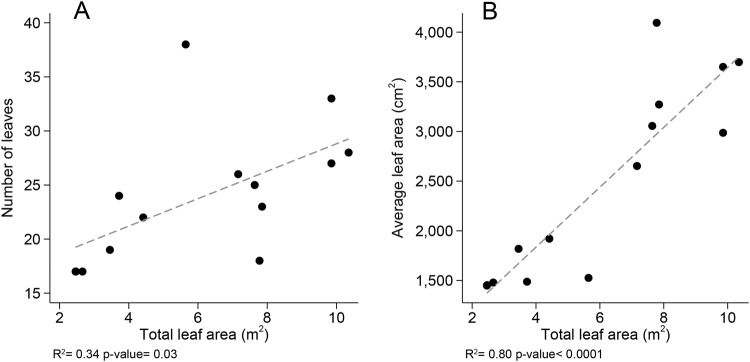
Leaf features contributing to total leaf area. (**A**) The relationship between number of leaves and total leaf area. (**B**) The relationship between average leaf area per leaf and total leaf area. The dashed lines represent linear regressions.

The *Cecropia* trees we sampled ranged from 2.42–7.95 m tall with 55–144 hollow internodes that provided a total internal volume of 0.23–5.65 L with an estimated age range of 1–4.5 years. Only the oldest tree bore inflorescences during the study (*C*. *insignis*, 2 inflorescences). Leaf area and tree height scaled with marginally significant negative allometry such that every 10-fold increase in height produced a 7-fold increase in leaf area (regression, r^2^ = 0.79, scaling coefficient (log-log slope) = 0.77, Wald test for comparing the scaling coefficient to 1, *p* = 0.073). Total leaf area was driven more by an increase in leaf size rather than leaf number (Fig. 2). Tree height and estimated age were not correlated (regression, n = 14, *p* = 0.47).

All colonies were identified as *A*. *constructor*, monogynous, and ranged in size from 1,880–13,534 workers, with 73–93% of the workforce on the external surface of their tree at the time of harvesting. Alate production was low, with only 2 of the larger colonies producing 1–22 males and no females. The number of brood and number of workers scaled with negative allometry such that with every 10-fold increase in workers, there was only a 4-fold increase in brood (regression, r^2^ = 0.22, scaling coefficient = 0.41, Wald test *p* = 0.019).

**Figure 3:**
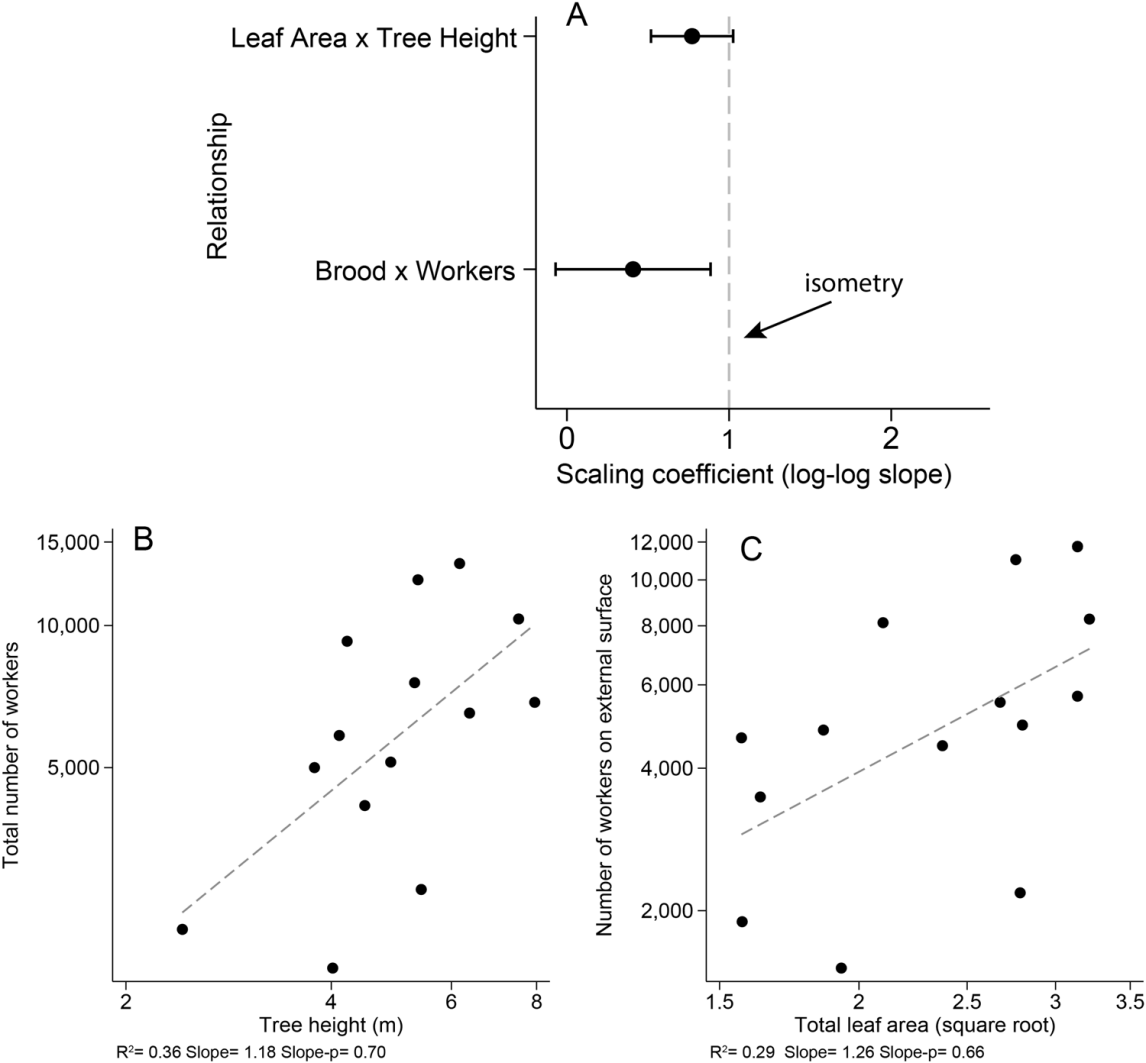
(**A**) A comparison of the scaling coefficients ± confidence intervals for the relationship between brood-to-workers and leaf area-to-tree height. (**B**) The relationship between total number of workers and tree height. The dashed line represents an allometric regression (log-log relationship). “Slope” indicates the observed scaling coefficient and “Slope-p” indicates the *p*-value resulting from a Wald test comparing the predicted and observed scaling coefficients. The slope of this line (the scaling coefficient) was not significantly different from 1, indicating an isometric relationship. (**C**) The relationship between the number of workers on the external surface of the plant and total leaf area. The dashed line represents an allometric regression (log-log relationship). The scaling coefficient was not significantly different from the 1, indicating an isometric relationship.

The scaling of brood-to-workers and leaf area-to-tree height was not significantly different, i.e., the log-log slope of number of brood vs. workers did not differ from the log-log slope of leaf area vs tree height (t-Value = 1.466, *p* = 0.155, Fig. 3A). The total number of workers scaled isometrically with tree height (regression, r^2^ = 0.36, scaling coefficient = 1.18, Wald test *p* = 0.70, Fig. 3B), meaning every 10-fold increase in tree height produces a 10-fold increase in the number of workers in the colony. Furthermore, the number of external workers increased isometrically with total leaf area (regression, r^2^ = 0.29, scaling coefficient = 1.26, Wald test *p* = 0.66, Fig. 3C) meaning the overall density of ants remains constant across the range we sampled. The total number of workers was not correlated with estimated tree age (correlation, n = 14, *p* = 0.918).

### Nest organization and vertical distribution

**Figure 4:**
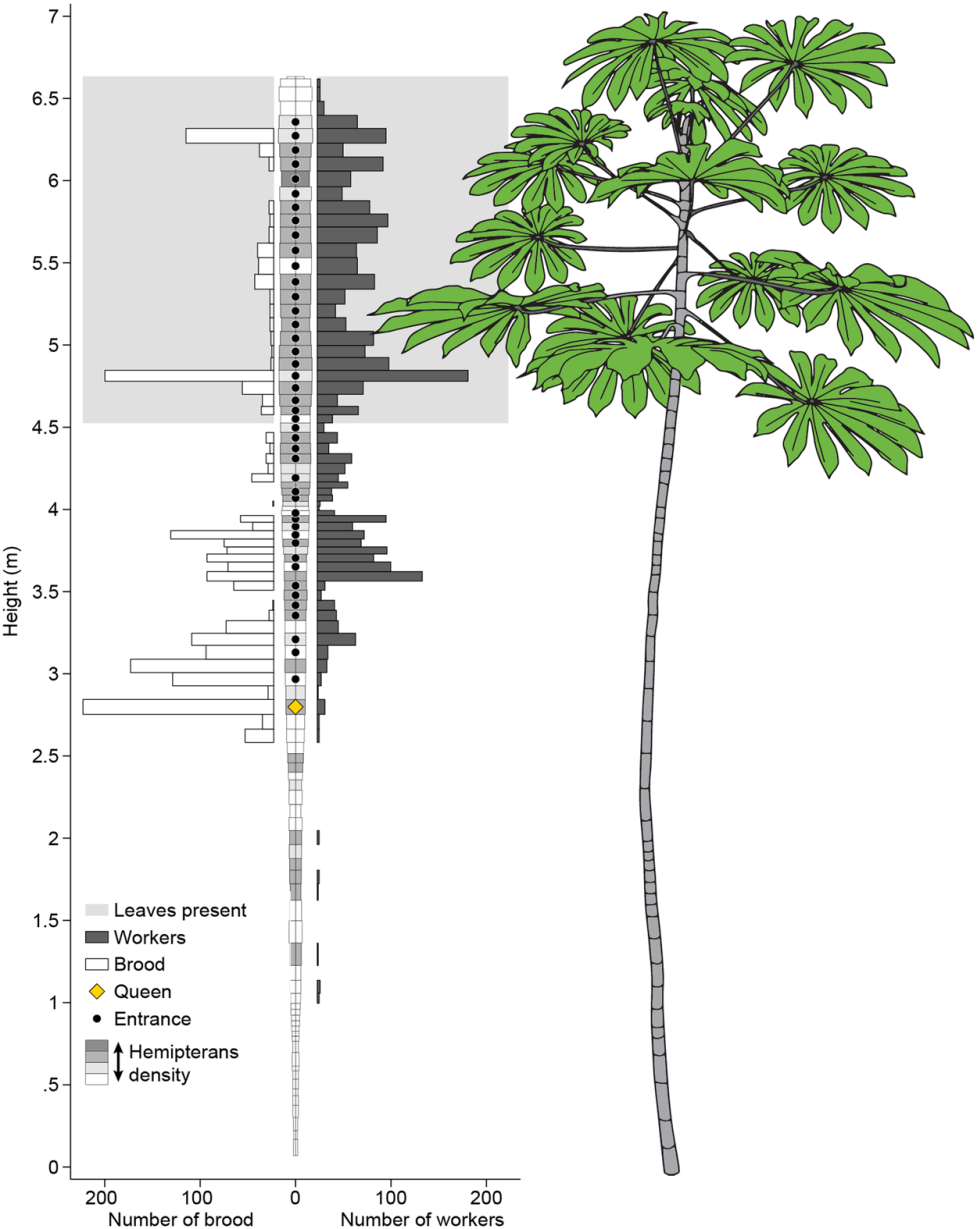
The distribution of colony nest components within an exemplar *Cecropia* tree. Each bar in the central column represents an internode from the central stem, and the dimensions are scaled to the height and width of the internal volume of each internode (width is doubled relative to height to show the components more clearly). The width of the bars to the left represent the number of brood and the bars to the right represent the number of workers. The shading of each internode indicates the hemipteran density. The shaded area near the top of the tree represents internodes that bore leaves. The location of the queen is indicated by the golden diamond, and entrances are indicated by black circles.

**Figure 5:**
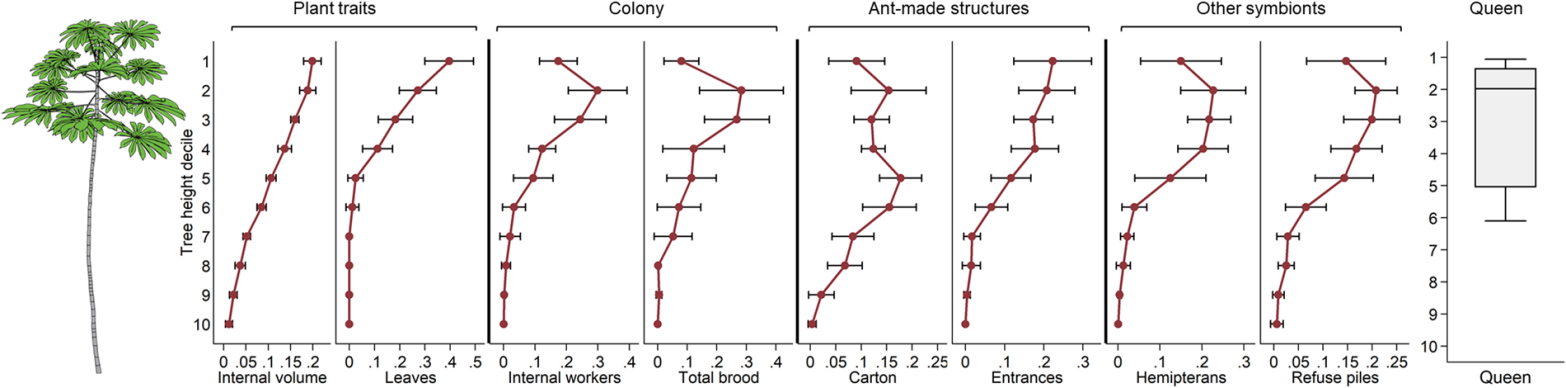
The mean proportion of each nest component as a function of tree height decile. Error bars indicate 95% confidence intervals. The box plot represents the decile where the queen was located.

**Figure 6:**
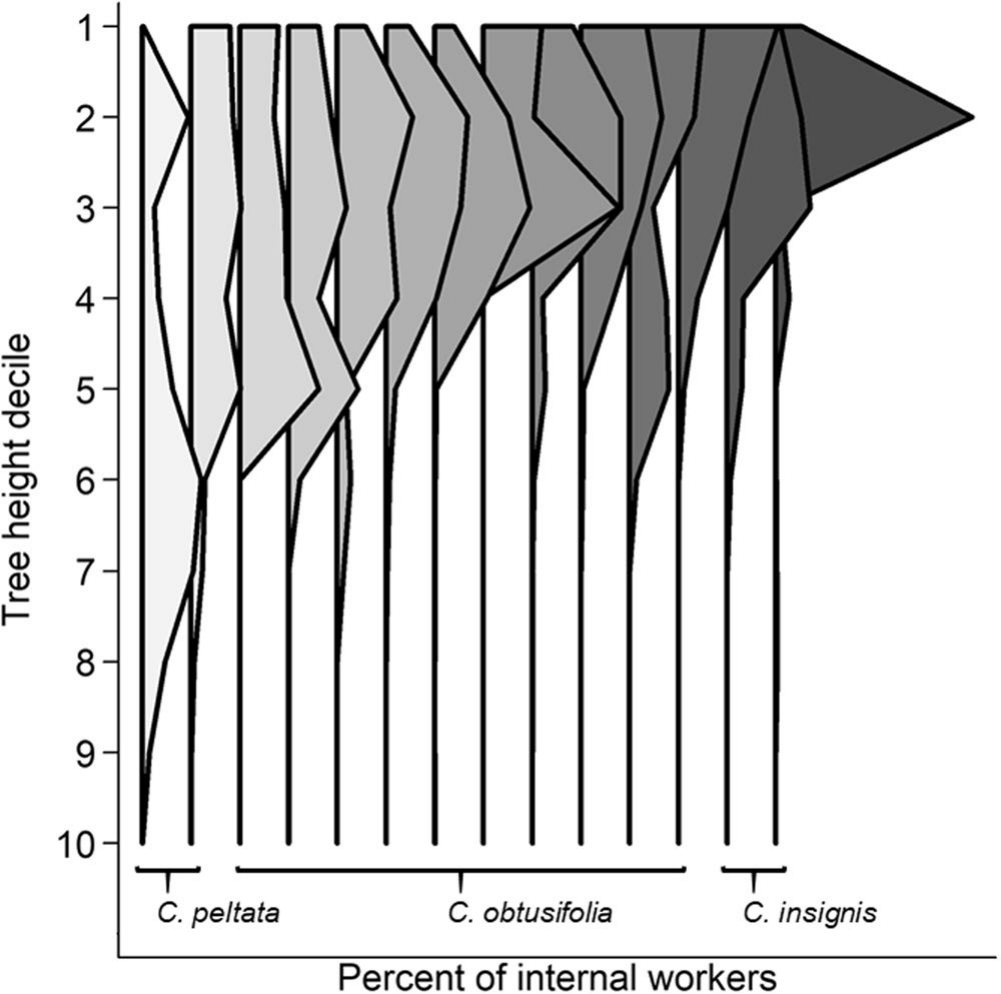
The distribution of workers within *Cecropia* trees. The proportion of internal workers are rendered by tree height decile for each tree. Colonies are arranged by *Cecropia* species, then by the proportional height of the median worker distribution. The proportional height of the median worker distribution differed significantly among *Cecropia* species (ANOVA, F = 7.17, *p* = 0.01).

We detailed nest structure and vertical distribution for an exemplar colony in Fig. 4. Colonies occupied 27–62% of the available internodes. While worker distribution was often patchy, nearly all the upper stem was inhabited. To compare vertical distribution patterns across different tree and colony sizes, we rendered the proportion of each nest component by tree height decile, i.e., in 10% increments starting at the top of the tree (Fig. 5). Internal tree volume was not evenly distributed vertically, but steadily increased with decile height because newer, herbaceous internodes are larger and more spacious. The internal dimensions of the internodes do not change, but woody growth slowly increases the external diameter so older, lower internodes have a much smaller internal space with the same external diameter. Nearly all leaves were in the top half of the tree, with leaf proportion steadily increasing with decile height therein. The proportion of workers, brood, scale insects, and refuse piles peaked around the second and third height decile. Carton was more evenly distributed, tapering off in the lowest deciles, while the proportion of entrances steadily increased with decile height. The vertical distribution of workers differed by the *Cecropia* species they inhabit (ANOVA for proportional height of median workers, *p* < 0.05, Fig. 6), with *C*. *peltata* supporting a low, broad distribution, *C*. *insignis* supporting a high, narrow distribution, and *C*. *obtusifolia* ranging between the other two.

**Figure 7:**
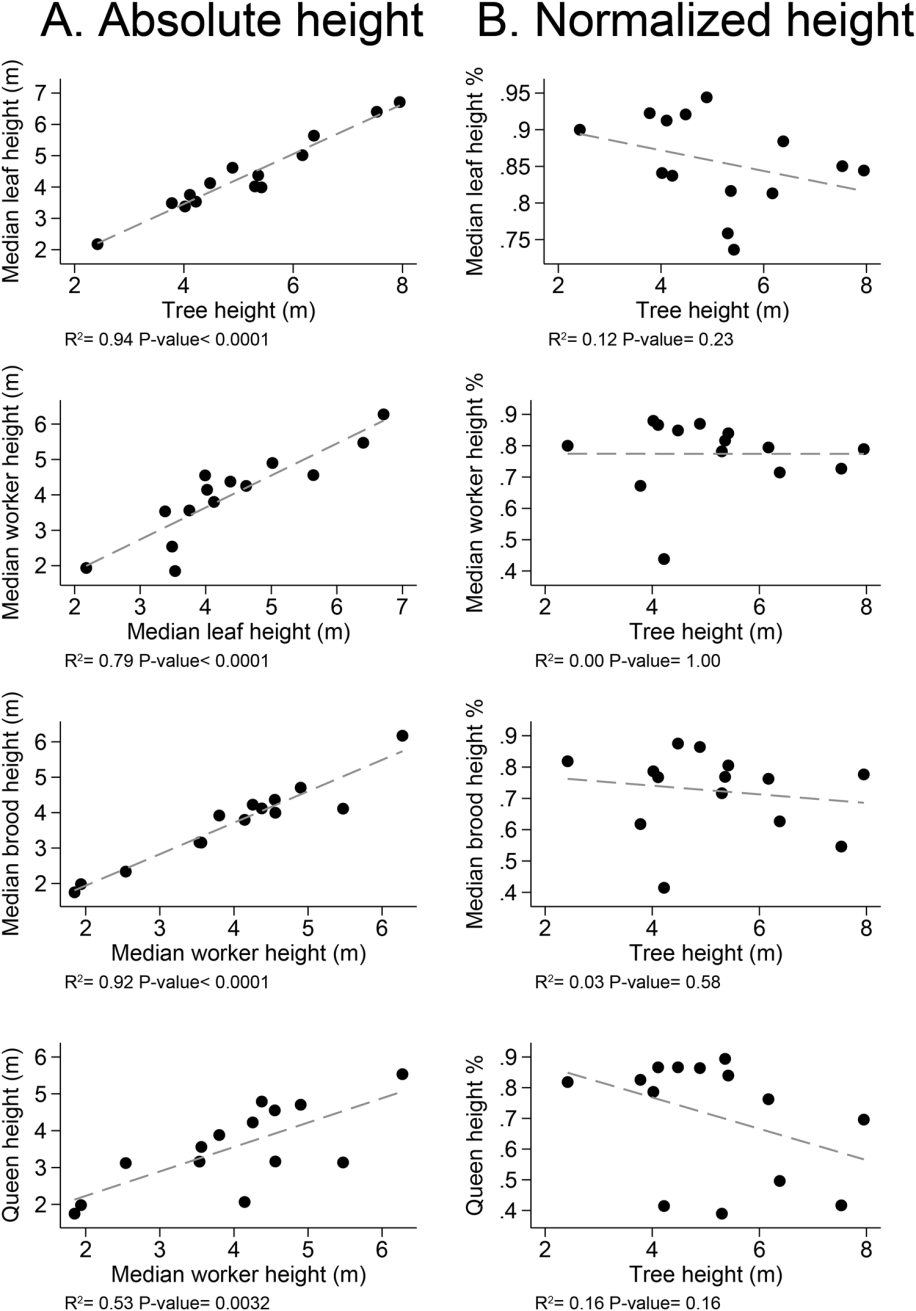
The relationship among the median height of tree and colony components. (**A**) The absolute height of each component. (**B**) The proportional height of each component relative to absolute tree height.

**Figure 8:**
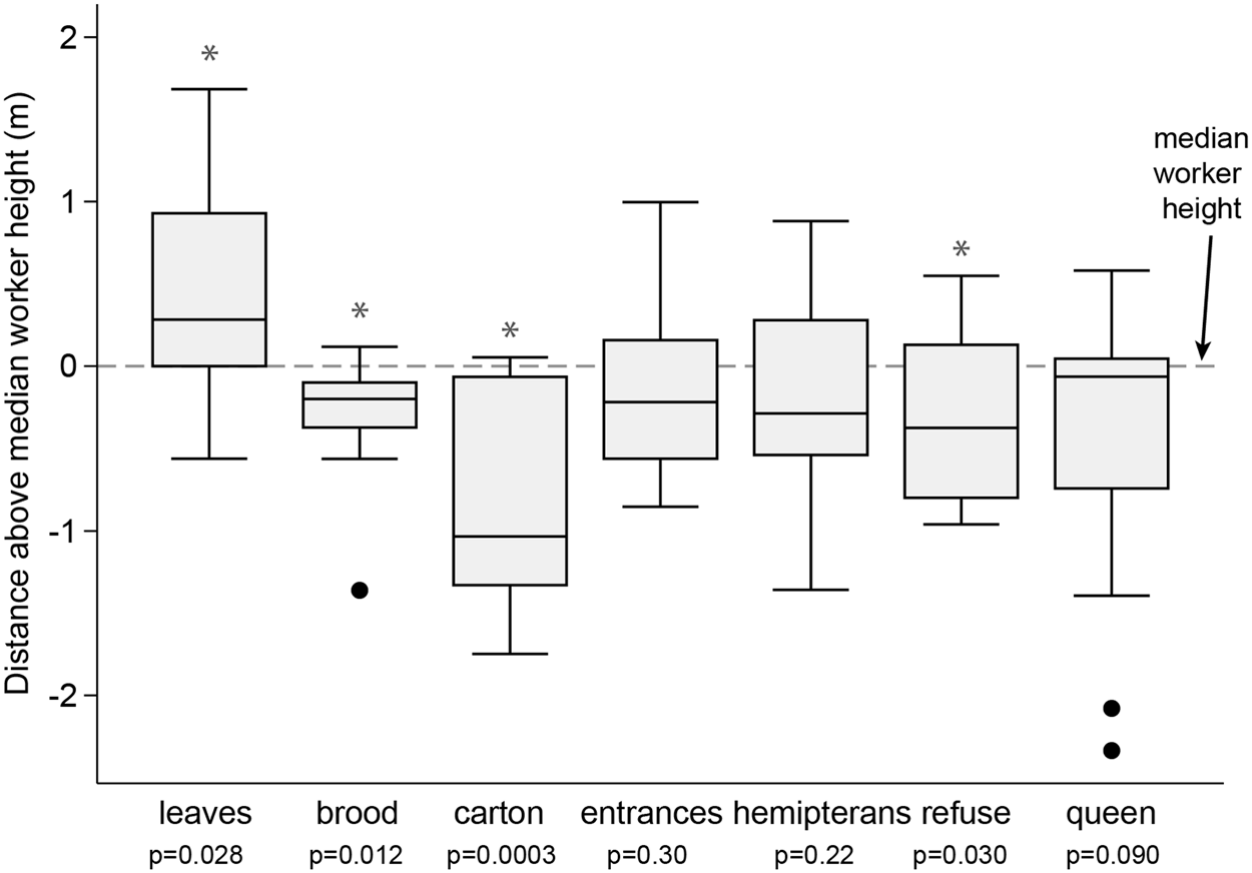
The distance in meters between median nest component heights and median worker height. The median height of workers is the height up the stem at which half of the internal ants reside above and half reside below. The same height was calculated for each nest component, e.g., the point at which half of the entrances are above and half are below. The distance in meters between the median worker height and each of the nest components were calculated for each tree and are represented here as box plots. Positive values indicate the median nest component was higher in the stem than the median worker height, while negative values indicate it was lower. An asterisk indicates a significant difference from the median worker height.

Nest component heights were correlated with tree and worker heights (Fig. 7A). The relative median height (percent of tree height) of these components is independent of tree height, i.e., the various tree components are at the same proportional location in the tree, regardless of the tree’s absolute height (Fig. 7B). Median worker distribution height was below median leaf height, but above brood, carton, and refuse median height. There was no difference between median worker height and scale insect height or queen height (paired t-test, Fig. 8).

While less than half of the total internodes contain carton (32 ± 4% mean ± s.e.), more than half of the total workers (66 ± 5%) and brood (82 ± 4%) reside in internodes with carton.

To determine the relationship among several nest components in individual ant-occupied internodes, we entered nest variables (the presence of entrances, queens, and carton, and the number of brood, scale insects, and refuse piles) for each internode of 14 trees (n = 1194 internodes total) into a principal component analysis. The first two principal components had eigenvalues greater than the mean and together explain 55% of the variation. Thus, each internode varied along two axes: a “resource management” score (PC1, entrance-hemipteran-refuse axis) and a “nursery” score (PC2, brood-queen-carton axis) (Table 1). Most internodes scored low on both, several scored high on one but not the other, and very few scored high on both.

**Table 1:** A summary of the principal component analysis for the nest components in each internode (n = 613). Dashes indicate loading scores below 0.2.

	PC1 “Resource management score”	PC2 “Nursery score”
Eigenvalue	1.72	1.57
Variance Explained	28.7%	26.2%
Loading Scores		
Number of brood	—	0.65
Number of refuse piles	0.61	—
Number of hemipterans	0.57	—
Entrance present	0.50	—
Carton present	—	0.46
Queen present	—	0.57

We analyzed how the royal chamber (the internode containing the queen) differed from other internodes by comparing the confidence intervals for the presence of each nest component (entrances, carton, brood, scale insects, and refuse piles) for all ant-occupied internodes to their presence in the royal chambers. Compared to an average ant-occupied internode, the royal chamber was more likely to contain carton and brood, and less likely to contain refuse piles. There was no significant difference for scale insect or entrances.

### Ant morphology

**Figure 9:**
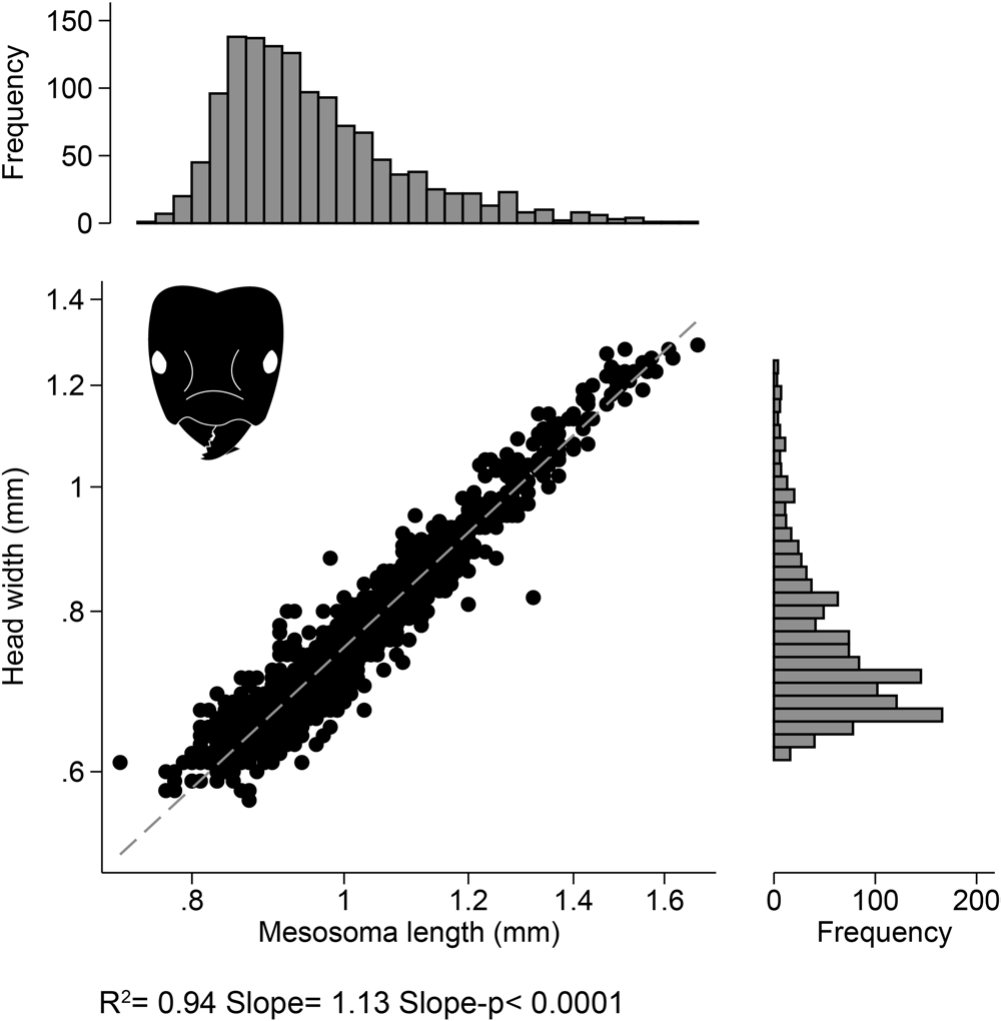
The relationship between head width and mesosoma length for workers from all colonies (n = 1,300). The dashed line represents an allometric regression (log-log relationship). The scaling coefficient was significantly higher than the predicted isometric slope of 1, indicating a positive allometric relationship. The histogram shows the frequency of workers by mesosoma length and head width on their respective axes.

Workers varied in size with head widths ranging from 0.57–1.29 mm and were positively allometric (workers from all colonies pooled together, log head width-log mesosoma length slope = 1.13, Fig. 9). To analyze variation among colonies in worker size, for each colony we calculated the mean worker head width, the maximum head width, the size range factor (max/min head width), and degree of allometry (log head-log mesosoma scaling coefficient). We report further PCA analysis on these measurements in the next section.

### Relationships among sociometric categories

Each of the five major categories of sociometrical data – tree size, colony size, nest structure, ant morphology, and colony personality – are complex with several variables, so we sought to simplify each category by an unrotated PCA. We then used the simplified descriptions to explore relationships among categories. For every PCA, the first principal component (PC1) had the only eigenvalue greater than the mean and explained a substantial majority of the variation. Furthermore, the nature of the loadings on PC1 were easily interpreted and given intuitive summary descriptors we outline below.

#### Tree size

Height, total internal volume, total leaf area, and stem diameter all loaded strongly positive and PC1 explained 90% (Table 2). We named PC1 “tree size” because higher values indicate taller trees with greater diameter, internal volume, and leaf area.

**Table 2:** A summary of the principal component analyses for the each sociometric categories – tree size, colony size, nest structure, and worker size.

	PC1 “Tree size”	PC2 (not used)
Eigenvalue	3.67	0.17
Variance Explained	91.8%	4.3%
Loading Scores		
Height	0.50	—
Diameter	0.50	0.74
Internal volume	0.49	—
Total leaf area	0.49	−0.65
	**PC1 “Colony size”**	**PC2 (not used)**
Eigenvalue	2.90	0.53
Variance Explained	72.6%	13.3%
Loading Scores		
Total workers	0.51	−0.28
Total brood	0.44	0.89
Number of refuse piles	0.51	—
Number of hemipterans	0.52	−0.30
	**PC1 “Colony distribution breadth”**	**PC2 (not used)**
Eigenvalue	3.71	0.67
Variance Explained	74.2%	13.2%
Loading Scores		
Percent of internodes with workers	−0.40	0.70
Percent of internodes with brood	−0.45	0.31
Median proportional height of workers	0.43	0.57
Median proportional height of workers	0.49	0.25
Proportional height of the queen	0.42	—
	**PC1 “Worker size”**	**PC2 (not used)**
Eigenvalue	3.21	0.45
Variance Explained	80.2%	11.3%
Loading Scores		
Mean head width	0.48	0.34
Max head width	0.52	−0.45
Size range factor (max head/min head width)	0.52	−0.46
Head-mesosoma scaling coefficient (log-log slope)	0.46	0.67
	**PC1 “Colony personality”**	**PC2 (not used)**
Eigenvalue	1.934	1.065
Variance Explained	48.3%	26.6%
Loading Scores		
Patrolling	0.620	0.236
Vibrational disturbance	0.351	0.731
Intruder response	0.511	0.262
Leaf Damage Response	0.482	−0.610

**Figure 10:**
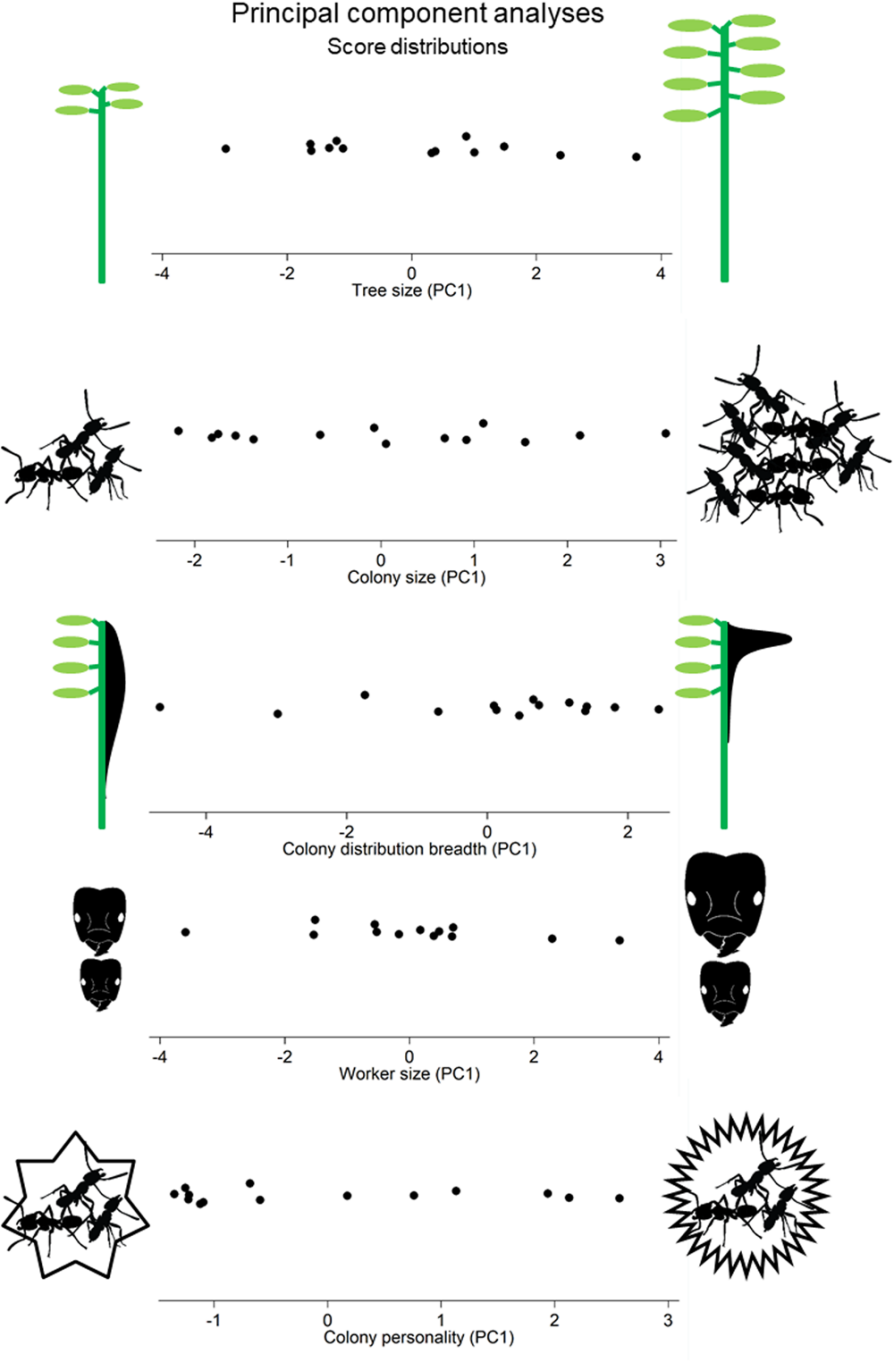
Score distributions for the 5 major sociometric categories. Plots display how colonies vary along the PC1 axes for tree size, colony size, colony distribution breadth, worker size, and colony personality. The illustrations on either side are visual interpretations of what the extreme values represent for each PC1. For colony personality, higher values indicate more active, aggressive colonies.

Dashes indicate loading scores below 0.2. The PCA data for colony personality is from^34^, but is included here for completeness. See Fig. 10 for a visualization of how colonies are distributed along each PC1.

#### Colony size

Total workers, brood, hemipterans, and refuse piles all loaded strongly positive and PC1 explained 71% (Table 2). We named PC1 “colony size” because higher values indicate colonies with more workers, brood, hemipterans, and refuse piles.

#### Colony distribution breadth

Queen, median worker, and median brood height loaded strongly positive, while the percent of total internodes with worker and brood present loaded strongly negative and PC1 explained 74% (Table 2). We named PC1 “colony distribution breadth” because higher values indicate that the colony nest components have narrower distribution and are located higher in the tree.

#### Worker size

Allometry slope, size range factor, max head width, and average head width all loaded strongly positive and PC1 explained 80% (Table 2). We named PC1 “worker size” because colonies with higher values have larger workers, greater size disparities, and steeper allometries.

#### Colony personality

The results for colony behavior were published in^34^, but we include them here for congruency (Table 2). Vibrational disturbance, leaf damage, intruder, and patrolling all loaded strongly positive and PC1 explained 48%. We named PC1 “colony personality” and colonies with higher values were more active, aggressive, and responsive.

**Figure 11:**
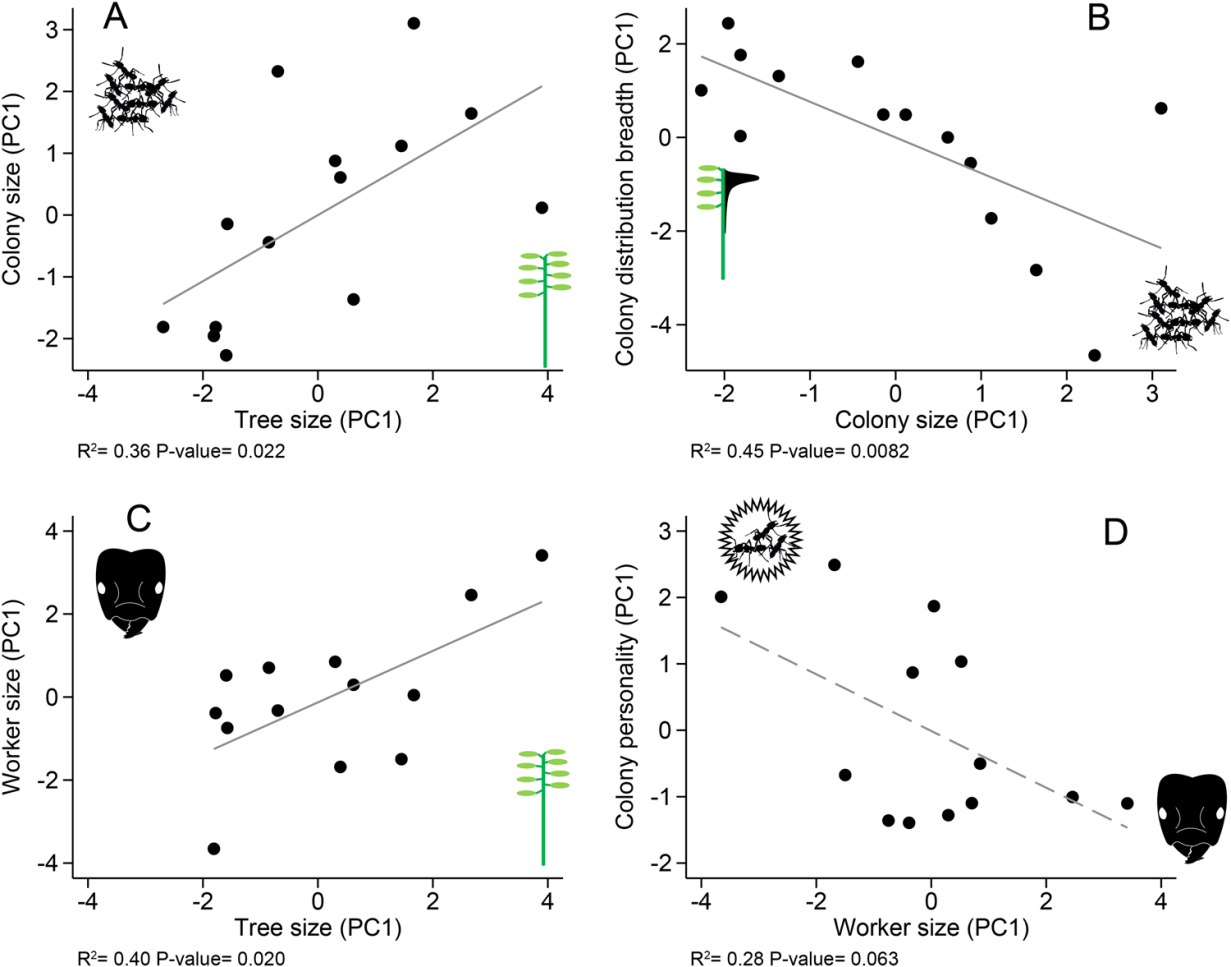
Correlations among sociometric categories. Solid lines indicate a significant correlation (*p* < 0.05) between traits and the dashed line indicates a nearly significant trend (*p* < 0.1). (**A**) The relationship between colony size and tree size. (**B**) the relationship between colony distribution breadth and colony size. (**C**) The relationship between worker size and tree size. (**D**) The relationship between colony personality and worker size.

The colony scores for PC1 of each sociometrical category are summarized in Fig. 10. We tested for correlations among all sociometrical categories using these PC1 scores (Table 2). Significant and trending correlations are shown in Fig. 11; larger trees supported larger colonies (*p* = 0.02, Fig. 11A), larger colonies promoted broader nest distributions (*p* = 0.008, Fig. 11B), larger trees supported larger, more allometric worker morphologies (*p* = 0.02, Fig. 11C), and colonies with larger, more allometric worker morphologies tended to be less aggressive (*p* = 0.06, Fig. 11D). There was no correlation between ant morphology and colony size (*p* = 0.47).

## Discussion

Our results support the notion that the growth, nest organization, and morphology of *Azteca constructor* colonies are intertwined with their *Cecropia* host plants. Costs to the host plant can accrue if tree growth outpaces colony growth^10–12^ or vice versa^16,17^, but our results show that, over the size range that we sampled, colony and plant growth rates are similar. Furthermore, colony size increased isometrically with tree height, but not with tree age. Older trees were not necessarily taller, which likely reflects that some plants are growing in unfavorable conditions, e.g., poor soil nutrients^43^ or low light^56^, which in turn likely affects colony growth. This provides further evidence that there is positive feedback between colony and plant growth rates that stabilizes the mutualism. Additionally, the number of workers on the external surfaces, i.e., the stem, leaves, and petioles, increased isometrically with total host plant leaf area, suggesting that ant density remains consistent as the tree grows. Leaf damage did not increase with tree size, as it does with *Cordia* plants^10^, but rather decreased with colony-level aggression^34^, suggesting that colony behavior is more important for preventing herbivory than colony size. For colonies to effectively reduce herbivory, they must successfully search leaves, communicate threats, and recruit workers appropriately. The optimal strategy for collective search and deployment may depend on threat level^57^, colony size^58^, or territory size and shape^59,60^. Given that individual leaf size increases with tree height (Fig. 2B), the most effective patrolling strategy may shift as the colony and the plant grow. Further research is merited to test whether colonies employ different collective search strategies as their host plant surfaces increase. Some plant-ants have evolved secondary polygyny as a possible solution to diminishing growth rates relative to their host plant^13,14^. However, the more synchronized growth rates in the *Azteca-Cecropia* system may negate the benefits of secondary polygyny, leading to the evolution of secondary monogyny instead^15^.

The spatial distribution of colonies within their hosts also follows tree structure. Vertical worker distribution tended to be most dense near the top of the tree, which reflects the distribution of available nesting space and food-body-bearing leaves. While we did not measure how ants were distributed among leaves themselves, previous work indicates that most ants occur in the upper third of the of the leaves despite most of the leaf area occurring in the middle third^61^. The overabundance of workers on younger, newer leaves reflects the contribution the leaf will make to plant growth^61^, which is likely driven by the fact that newer leaves produce the most food bodies^43^. The median leaf height was consistently above the median worker height in the internal stem, and median brood height was below the median worker height. This suggests that as new nesting space and leaves grow from the apical meristem, workers follow, then brood. Even though less than half of internodes contained carton galleries, we found that the majority of workers and most of the brood resided in internodes with carton, suggesting they serve as brood storage. The shape of vertical *Azteca* worker distributions resembled the distribution patterns of several ground-nesting ant species^21,62^, which may reflect comparable resource proximity or available nest volume.

Given the distribution shape and height of each nest component, we posit a generalized hypothesis about how the colony distributes itself as the tree grows. As trees grow upward, adding new leaves and larger internodes, workers quickly chew entrances and move into the new space, harvest the new food bodies, and bring the scale insects to feed on the softer tissues. Carton is built more slowly and eventually brood is deposited there. Lower internodes are eventually abandoned, leaving behind used carton and sealed entrances (workers must actively maintain the entrance sites by chewing, or the tree will eventually seal them, PRM, pers. obs.). This hypothesis is limited to the range of tree sizes included in this study. It appears that colony distribution patterns may shift dramatically as tree’s central stem bifurcates into several branching points. In the dissection of a larger tree in Costa Rica, the *A*. *constructor* colony distribution appeared to be very centralized, with the queen and all brood residing in a large, permanent, carton-filled bulge near the center of the tree^19^. Such a centralized configuration may be advantageous for workers patrolling and foraging across several distributed meristems. Future sampling should include a larger range of tree sizes and structures to capture the transition from a more vertically distributed to a more centralized nest structure.

Despite the generalized pattern, there was a large amount of variation in how colonies distributed themselves within their trees. This variation is partially explained by larger colonies having broader distributions, but other factors not measured here may influence colony distribution. In *Temnothorax* ants, colonies consistently vary in how they structure their nests across time and contexts^63^. Our data were snapshots of colony distribution – it would be interesting to test whether patterns of colony distribution are consistent across time or persist across host plant transplants.

The close association between tree growth and colony structure extends to worker size. In many ant species where workers vary in size, worker morphology correlates with colony size and age, with larger colonies producing larger workers, greater size variation, and steeper allometries^24^. This trend reflects the natural progression of resource acquisition, colony nutrition, and colony growth. Intriguingly, here we show that worker size is not correlated with colony size or age, but rather host tree size. Worker morphology may be controlled by intrinsic factors like nutrition; larger trees may produce more food bodies, more nutrition is invested per larvae, resulting in larger workers. It is also possible that the nutrient ratios of the food bodies shift with tree height, resulting in larger workers. Worker size may also be responding to external factors like available space, load size, or entrance size. Larger trees naturally provide more voluminous chambers, greater surface area, and larger territory to patrol, which could be more efficiently traversed by larger workers. Perhaps the size of individual food bodies increases with tree size and are more efficiently carried by larger workers. Finally, larger trees may have larger prostomas – the dedicated dimpled sites where ants chew entrances into the internal internode space. Larger-headed workers may fill larger entrance gaps more appropriately to prevent intruders from entering the tree as in turtle ants^64^.

Colony personality was independent of colony size, tree size, and vertical distribution. However, an interesting pattern may emerge with ant morphology. Colonies with more aggressive personalities *tended* to have smaller, less allometric worker morphologies, which contradicts our hypothesis. Although the trend was weak, it is potentially interesting and worth more exploration. The trend may reflect some resource investment tradeoff between collective aggression and worker size – perhaps colonies can either have an aggressive demeanor or larger workers, but not both. Alternatively, worker size may be connected to task demand. Our measures of aggression are based on the number of ants responding to a given stimulus. If the colony has larger workers, perhaps fewer ants need to respond because they are more efficient at dealing with threats. A third possibility is that colonies fed more food bodies can produce larger workers than colonies not fed enough food bodies. Colonies not fed enough may try to compensate for their nutrient deficiency by increasing prey consumption^65^, thus resulting in a more aggressive collective personality. More experiments are needed to tease apart the correlation between worker size, colony personality, and tree size, as well as a proper foodweb analysis.

Food body production likely plays an important role in ant-plant sociometry, and therefore our view is limited by the fact that we were unable to quantify food rewards in this study. Food body production not only depends on ontogenetic factors we measured like plant height and leaf area^12^, but also environmental factors like soil nutrients^43,66^ and light availability^56^. It would be interesting to test how these factors contribute not only to the number and mass of food bodies, but how the nutrient content and size of individual food bodies might change as the plant grows. Food body production likely influences many aspects of ant sociometry, such as colony size^67^, distribution on leaves^61^, worker size, and colony behavior. Our study provides a good foundation to further test hypotheses about how food rewards fit in.

Our study on ant-plant sociometry is a comprehensive investigation on growth scaling, colony organization and vertical distribution, worker morphology, and collective personality in an ant-plant mutualism. We show the synchronization of plant growth and colony growth in the *Azteca-Cecropia* mutualism, a novel finding that supports the idea that such synchronization is a crucial enabler of the stability of a mutualism. *Azteca* sociometry is intimately intertwined with host plant biology and is an important consideration for mutualism dynamics. Our study may be valuable for the interpretation of other mutualisms between plants and stem-nesting ants, shedding light on convergent evolution and the unique strategies of these fascinating symbioses.

## Data Availability

The data associated with this manuscript have been deposited at Dryad Digital Repository (doi link will be provided).

## References

[1] Tschinkel WR (1991). Insect sociometry, a field in search of data. Insectes Soc..

[2] Bischof S (2013). *Cecropia peltata* accumulates starch or soluble glycogen by differentially regulating starch biosynthetic genes. Plant Cell.

[3] Rickson FR (1971). Glycogen plastids in Müllerian body cells of *Cecropia peltata* - a higher green plant. Science.

[4] Schupp EW (1986). *Azteca* protection of *Cecropia*: Ant occupation benefits juvenile trees. Oecologia.

[5] Janzen DH (1969). Allelopathy by myrmecophytes: The ant *Azteca* as an allelopathic agent of *Cecropia*. Ecology.

[6] Dejean A, Petitclerc F, Roux O, Orivel J, Leroy C (2012). Does exogenic food benefit both partners in an ant-plant mutualism? The case of *Cecropia obtusa* and its guest *Azteca* plant-ants. Comptes Rendus - Biol..

[7] Oliveira KN (2015). The effect of symbiotic ant colonies on plant growth: A test using an *Azteca-Cecropia* system. PLoS One.

[8] Sagers CL, Ginger SM, Evans RD (2000). Carbon and nitrogen isotopes trace nutrient exchange in an ant plant mutualism. Oecologia.

[9] Gutiérrez-Valencia J, Chomicki G, Renner SS (2017). Recurrent breakdowns of mutualisms with ants in the neotropical ant-plant genus *Cecropia* (Urticaceae). Mol. Phylogenet. Evol..

[10] Pringle EG, Dirzo R, Gordon DM (2012). Plant defense, herbivory, and the growth of *Cordia alliodora* trees and their symbiotic *Azteca* ant colonies. Oecologia.

[11] Handa C (2013). Change in biomass of symbiotic ants throughout the ontogeny of a myrmecophyte, *Macaranga beccariana* (Euphorbiaceae). J. Plant Res..

[12] Heil M (1997). Food body production in *Macaranga triloba* (Euphorbiaceae): A plant investment in anti-herbivore defence via symbiotic ant partners. J. Ecol..

[13] Feldhaar H, Fiala B, Bin Hashim R, Maschwitz U (2000). Maintaining an ant-plant symbiosis: Secondary polygyny in the *Macaranga triloba-Crematogaster* sp. association. Naturwissenschaften.

[14] Kautz S, Pauls SU, Ballhorn DJ, Lumbsch HT, Heil M (2009). Polygynous supercolonies of the acacia-ant *Pseudomyrmex peperi*, an inferior colony founder. Mol. Ecol..

[15] Perlman, D. L. *Colony founding among* Azteca *ants*. PhD dissertation, Harvard University, Department of Organismic and Evolutionary Biology (1992).

[16] Fonseca CR (1993). Nesting space limits colony size of the plant-ant *Pseudomyrmex concolor*. Oikos.

[17] Fonseca CR (1999). Amazonian ant-plant interactions and the nesting space limitation hypothesis. J. Trop. Ecol..

[18] Frederickson ME, Gordon DM (2009). The intertwined population biology of two Amazonian myrmecophytes and their symbiotic ants. Ecology.

[19] Longino, J. T. *Azteca* ants in *Cecropia* trees: taxonomy, colony structure, and behaviour. In *Ant-plant interactions* (eds. Huxley, C. R. & Cutler, D. F.) 271–288 (Oxford University Press 1991).

[20] Tschinkel WR (1993). Sociometry and sociogenesis of colonies of the fire ant *Solenopsis invicta* during one annual cycle. Ecol. Monogr..

[21] Murdock TC, Tschinkel WR (2015). The life history and seasonal cycle of the ant, *Pheidole morrisi* Forel, as revealed by wax casting. Insectes Soc..

[22] Tschinkel WR (1988). Colony growth and the ontogeny of worker polymorphism in the fire ant. Solenopsis invicta. Behav. Ecol. Sociobiol..

[23] Kwapich CL, Gadau J, Hölldobler B (2017). The ecological and genetic basis of annual worker production in the desert seed harvesting ant. Veromessor pergandei. Behav. Ecol. Sociobiol..

[24] Wills BD, Powell S, Rivera MD, Suarez AV (2018). Correlates and consequences of worker polymorphism in ants. Annu. Rev. Entomol..

[25] Chamberlain SA, Holland JN (2009). Body size predicts degree in ant-plant mutualistic networks. Funct. Ecol..

[26] Meunier L, Dalecky A, Berticat C, Gaume L, McKey D (1999). Worker size variation and the evolution of an ant-plant mutualism: Comparative morphometrics of workers of two closely related plant-ants, *Petalomyrmex phylax* and *Aphomomyrmex afer* (Formicinae). Insectes Soc..

[27] Gaume L, McKey D, Anstett MC (1997). Benefits conferred by ‘timid’ ants: Active anti-herbivore protection of the rainforest tree *Leonardoxa africana* by the minute ant *Petalomyrmex phylax*. Oecologia.

[28] Valverde JP, Hanson P (2011). Parenchyma: A neglected plant tissue in the *Cecropia*/ant mutualism. Symbiosis.

[29] Mayer VE (2018). Transmission of fungal partners to incipient *Cecropia*-tree ant colonies. PLoS One.

[30] Adams ES (1994). Territory defense by the ant *Azteca trigona*: maintenance of an arboreal ant mosaic. Oecologia.

[31] Bengston SE, Dornhaus A (2014). Be meek or be bold? A colony-level behavioural syndrome in ants. Proc. R. Soc. B Biol. Sci..

[32] Pruitt JN, Grinsted L, Settepani V (2013). Linking levels of personality: Personalities of the ‘average’ and ‘most extreme’ group members predict colony-level personality. Anim. Behav..

[33] Modlmeier AP, Foitzik S (2011). Productivity increases with variation in aggression among group members in *Temnothorax* ants. Behav. Ecol..

[34] Marting PR, Wcislo WT, Pratt SC (2018). Colony personality and plant health in the *Azteca-Cecropia* mutualism. Behav. Ecol..

[35] Wray MK, Mattila HR, Seeley TD (2011). Collective personalities in honeybee colonies are linked to colony fitness. Anim. Behav..

[36] Blight O, Villalta I, Cerdá X, Boulay R (2016). Personality traits are associated with colony productivity in the gypsy ant *Aphaenogaster senilis*. Behav. Ecol. Sociobiol..

[37] Modlmeier AP, Liebmann JE, Foitzik S (2012). Diverse societies are more productive: a lesson from ants. Proc. Biol. Sci..

[38] Wright CM, Keiser CN, Pruitt JN (2015). Personality and morphology shape task participation, collective foraging and escape behaviour in the social spider *Stegodyphus dumicola*. Anim. Behav..

[39] Bengston, S. E. *et al*. Genomic tools for behavioural ecologists to understand repeatable individual differences in behaviour. *Nat*. *Ecol*. *Evol*. 1–12 (2018). 10.1038/s41559-017-0411-4 PMC9437744 29434349

[40] Sih A (2015). Animal personality and state-behaviour feedbacks: A review and guide for empiricists. Trends Ecol. Evol..

[41] Pinter-Wollman N (2015). Nest architecture shapes the collective behaviour of harvester ants. Biol. Lett..

[42] Burd M, Shiwakoti N, Sarvi M, Rose G (2010). Nest architecture and traffic flow: Large potential effects from small structural features. Ecol. Entomol..

[43] Folgarait PJ, Davidson DW (1995). Myrmecophytic *Cecropia*: antiherbivore defenses under different nutrient treatments. Oecologia.

[44] Biro PA, Stamps JA (2010). Do consistent individual differences in metabolic rate promote consistent individual differences in behavior?. Trends Ecol. Evol..

[45] Nowbahari E, Fénéron R, Malherbe M-C (1999). Effect of body size on aggression in the ant, *Cataglyphis niger* (Hymenoptera; Formicidae). Aggress. Behav..

[46] Berg, C. C., Rosselli, P. F. & Davidson, D. W. *Cecropia*. (New York Botanical Garden Press, Flora Neotropica 2005).

[47] Sposito TC, Santos FAM (2001). Scaling of stem and crown in eight *Cecropia* (Cecropiaceae) species of Brazil. Am. J. Bot..

[48] Zalamea PC, Stevenson PR, Madriñán S, Aubert PM, Heuret P (2008). Growth pattern and age determination for *Cecropia sciadophylla* (Urticaceae). Am. J. Bot..

[49] Alvarez-Buylla ER, Martinez-Ramos M (1992). Demography and allometry of Cecropia Obtusifolia, a Neotropical pioneer tree - an evaluation of the climax-pioneer paradigm for tropical rain forests. J. Ecol..

[50] Longino JT (1991). Taxonomy of the *Cecropia* inhabiting *Azteca* ants. J. Nat. Hist..

[51] Mayer VE, Voglmayr H (2009). Mycelial carton galleries of *Azteca brevis* (Formicidae) as a multi-species network. Proc. R. Soc. B Biol. Sci..

[52] Esquivel A, Abolafia J, Hanson P, Pinto A (2012). A new species of nematode, *Sclerorhabditis neotropicalis* sp. n (Rhabditida), associated with *Azteca* ants in *Cecropia obtusifolia*. Nematropica.

[53] Nepel M (2016). Ant-cultivated Chaetothyriales in hollow stems of myrmecophytic *Cecropia* sp. trees - diversity and patterns. Fungal Ecol..

[54] Zalamea PC (2012). The genus *Cecropia*: A biological clock to estimate the age of recently disturbed areas in the neotropics. PLoS One.

[55] Jackson DA (1993). Stopping rules in principal components analysis: A comparison of heuristical and statistical approaches. Ecology.

[56] Folgarait PJ, Davidson DW (1994). Antiherbivore defenses of myrmecophytic *Cecropia* under different light regimes. Oikos.

[57] Powell S, Donaldson-Matasci M, Woodrow-Tomizuka A, Dornhaus A (2017). Context-dependent defences in turtle ants: Resource defensibility and threat level induce dynamic shifts in soldier deployment. Funct. Ecol..

[58] Dornhaus A, Powell S, Bengston S (2012). Group size and its effects on collective organization. Annu. Rev. Entomol..

[59] Adler FR, Gordon DM (1992). Information collection and spread by networks of patrolling ants. Am. Nat..

[60] Gordon DM (2017). Local regulation of trail networks of the arboreal turtle ant, Cephalotes goniodontus. Am. Nat..

[61] Downhower JF (1975). The distribution of ants on *Cecropia* leaves. Biotropica.

[62] Tschinkel WR, Hanley N (2017). Vertical organization of the division of labor within nests of the Florida harvester ant, *Pogonomyrmex badius*. PLoS One.

[63] DiRienzo N, Dornhaus A (2017). *Temnothorax rugatulus* ant colonies consistently vary in nest structure across time and context. PLoS One.

[64] Powell S (2008). Ecological specialization and the evolution of a specialized caste in *Cephalotes* ants. Funct. Ecol..

[65] Dejean A, Grangier J, Leroy C, Orivel J (2009). Predation and aggressiveness in host plant protection: a generalization using ants from the genus *Azteca*. Naturwissenschaften.

[66] Heil M (2002). Nutrient allocation of *Macaranga triloba* ant plants to growth, photosynthesis and indirect defence. Funct. Ecol..

[67] Heil M, Hilpert A, Fiala B (2001). & Eduard Linsenmair, K. Nutrient availability and indirect (biotic) defence in a Malaysian ant-plant. Oecologia.

